# A Set of Platforms with Combinatorial and High-Throughput Technique for Gas Sensing, from Material to Device and to System

**DOI:** 10.3390/mi9110606

**Published:** 2018-11-19

**Authors:** Zhenghao Mao, Jianchao Wang, Youjin Gong, Heng Yang, Shunping Zhang

**Affiliations:** 1 Institute of Nuclear Physics and Chemistry, China Academy of Engineering Physics, Mianyang 621900, China; zh.mao@foxmail.com (Z.M.); gyjlyq@163.com (Y.G.); 2 Nanomaterials and Smart Sensors Research Laboratory, Department of Materials Science and Engineering, Huazhong University of Science and Technology, Wuhan 430074, China; 15827258559@163.com (J.W.); yy9512812@163.com (H.Y.); 3 Shenzhen Institute of Huazhong University of Science & Technology, Shenzhen 518000, China

**Keywords:** combinatorial and high-throughput technique, array optimization, electronic nose, efficiency

## Abstract

In a new E-nose development, the sensor array needs to be optimized to have enough sensitivity and selectivity for gas/odor classification in the application. The development process includes the preparation of gas sensitive materials, gas sensor fabrication, array optimization, sensor array package and E-nose system integration, which would take a long time to complete. A set of platforms including a gas sensing film parallel synthesis platform, high-throughput gas sensing unmanned testing platform and a handheld wireless E-nose system were presented in this paper to improve the efficiency of a new E-nose development. Inkjet printing was used to parallel synthesize sensor libraries (400 sensors can be prepared each time). For gas sensor selection and array optimization, a high-throughput unmanned testing platform was designed and fabricated for gas sensing measurements of more than 1000 materials synchronously. The structures of a handheld wireless E-nose system with low power were presented in detail. Using the proposed hardware platforms, a new E-nose development might only take one week.

## 1. Introduction

The electronic nose (E-nose) is an analytical device that plays a constantly growing role as a general purpose detector of vapor chemicals in many applications such as the quality control of the food industry [1,2], environmental protection [3,4,5], public safety [6] and spaceflight applications [7], according to articles on the subject that have been published over the last fifteen years. The core component in E-noses is the gas sensor array, which is made up of several gas sensors with different gas sensing properties to improve selectivity to gas/odor [8,9]. For a specific E-nose application, the gas sensor array needs to be optimized to the appropriate size and components [10,11,12]. There are several essential processes in E-nose development: the preparation of gas sensitive materials, gas sensor fabrication, array optimization, sensor array package and E-nose system integration. All these processes have an impact on the properties of sensitivity, selectivity, stability, price and power of the E-nose system [13]. Aside from the 3S2P (Sensitivity, Selectivity, Stability, Price and Power) properties, another factor that also needs to be considered is “Efficiency.” In other words, how long these processes would take to develop a new application, one month or one week?

For a new application, it takes a lot of time to find the optimized sensor array. The optimized sensor array could be selected from a sensor library that contains many gas sensors with different sensing properties [10,11,12]. The gas sensitive materials of these sensors could be synthesized through many methods, for example, a metal oxide semiconductor could be used as a gas sensitive material such as SnO_2_, ZnO, WO_3_ and In_2_O_3_ [14,15,16,17,18]. However, it would take a long time to fabricate the sensor library. The gas sensing properties of these sensors need to be measured according to the gas/odor to be classified in the E-nose application, which also takes a long time. After optimizing the sensor array, the E-nose system needs to be integrated based on the optimized sensor array and application. The E-nose system’s measuring range of sensor signals should be large enough to optimize the array. For gases/odor identification, the knowledge database of the E-nose system needs to be comprehensive to the optimized array and the gases/odor in the application. Accordingly, to improve the “Efficiency” in E-nose development, three processes should be considered: material parallel synthesis, high throughput screening and general utility of the E-nose system. 

Parallel synthesis and high throughput screening (called combinatorial and high-throughput technique) of gas sensitive materials could accelerate the selection of gas sensitive materials with good sensitivity, selectivity and stability. It could also be used in sensor array optimization. The difference between using the combinatorial method in materials selection and array optimization is the parallel synthesizing of gas sensitive materials or gas sensing films of sensor devices. For example, the Simon group used sol-gel and polyol methods to parallel synthesize gas sensitive materials [19,20,21,22]. Sol-gel and polyol methods are material synthesis methods and may not be suitable for gas sensing film synthesis in the batch production of sensor devices. The traditional thick film forming method of gas sensor fabrication is screen printing, however, it takes a long time to prepare the paste for screen printing and only one sensing film can be printed at a time.

In this paper, the inkjet printing technique and a high throughput gas sensing unmanned testing platform for the signal measurement of the sensor library were presented. After gas sensing films were parallel synthesized on the substrate, the sensor array (substrate with sensing films) needs to be packaged into a gas sensor for the acquisition of further performance. A simple sensor array package structure with low power and low price was also detailed in this paper. A handheld wireless E-nose system with the ability to measure 100 Ω to 1 GΩ sensor resistance was also described. With the above platforms, the sensor array could be easily fabricated, optimized, packaged and assembled into an E-nose system for a new E-nose application within one week.

## 2. Combinatorial and High-Throughput Technique for Screening Gas Sensor 

### 2.1. Parallel Synthesis of Sensor Library

The application of a combinatorial and high-throughput technique in materials research promises significant acceleration, especially in the area of material and parameter optimization as well as in the discovery of new materials. The first and significant step of the combinatorial and high-throughput technique is the parallel synthesis of different gas sensitive materials. The combinatorial and high-throughput technique could also be used in gas sensor selection and array optimization. The first step may not be the parallel synthesis of different materials but the parallel synthesis of different gas sensing films, which could be used in the batch production of sensor devices. This is because the optimized array is a sensor device for batch production and E-nose application. Many parallel synthesis methods of gas sensitive materials such as sol-gel and polyol methods could not be directly used to parallel synthesize gas sensing films in gas sensors.

**Figure 1:**
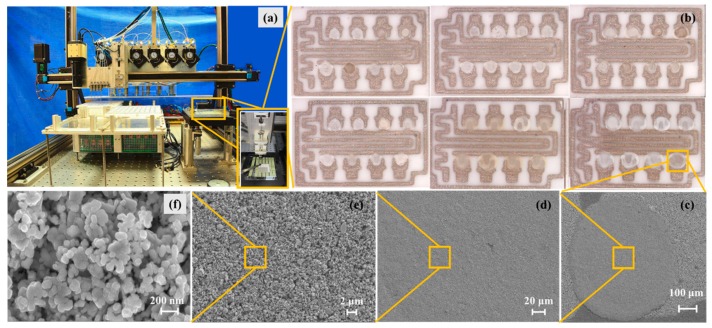
The parallel synthesis of gas sensing films. (**a**) The gas sensing film parallel synthesis platform. (**b**) The 8-matrices material substrate with sensing films. (**c**–**f**) Different magnifications of one gas sensing film.

**Figure 2:**
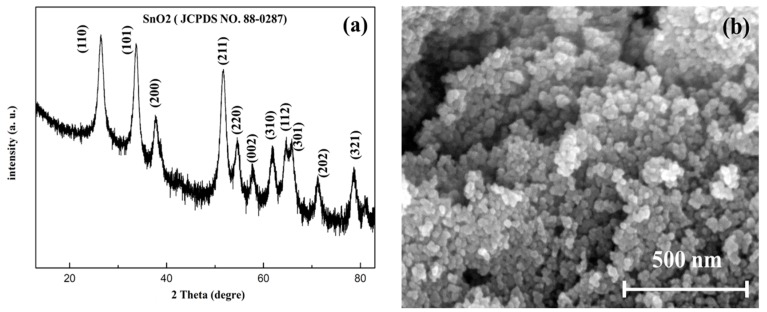
The X-ray diffraction (XRD) and scanning electron microscopy (SEM) images of the raw material. (**a**) X-ray diffraction pattern of SnO_2_ nanoparticles. (**b**) SEM image of SnO_2_ nanoparticles.

**Figure 3:**
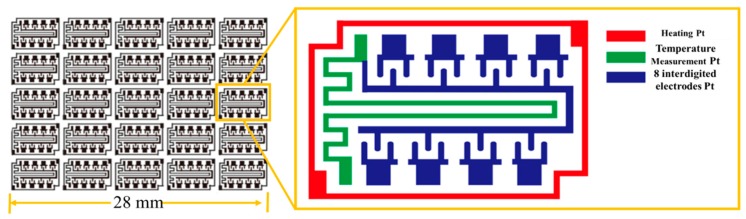
The structure of the 8-matrices material substrate.

For the parallel synthesis of gas sensing films, a platform named the gas sensing film parallel synthesis platform (shown in Figure 1a) was designed and manufactured. The platform consisted of two main parts: the premix module and the transfer printing module. The premix module consisted of a raw material cavity, peristaltic pump array, droplet needle array, premixed chamber, blender array and so forth. The transfer printing module was composed of the transfer printing needle and deflection angle camera. The gas sensing film parallel synthesis platform is suitable for metal oxide gas sensing materials. Here, the experiment of precious metal ions modified SnO_2_ is shown as an example to introduce the platform. As can be seen from Figure 2a, the SnO_2_ peaks fit the rutile structure and no secondary phase was detected. All peaks indexed well to the SnO_2_ JCPDS (Joint Committee on Poder Diffraction Standards) No. 88-0287. The high resolution transmission electron microscopy (HR-TEM; Tecnai G^2^ F30, FEI Company, Eindhoven, Netherlands) image of pure SnO_2_ is exhibited in Figure 2b where the particle size is approximately 15 nm. After preparing the gas sensitive material, jettable inks were made by dispersing SnO_2_ into deionized water with ball milling and were collected in the raw material cavity. Then, premixed solutions with different components conserved in the premixed chamber were obtained by mixing SnO_2_ inks and various additives (precious metal ionic solution) with the peristaltic pump and droplet needle array. To ensure the homogeneity of the premixed solutions, an array blender was used. A deflection angle camera was used to calibrate the deflection of the 8-matrices material substrate shown in Figure 3. The transfer printing needle was utilized to deposit the gas sensing films by transferring the premixed solutions to the 8-matrices material substrate. Finally, the 8-matrices material substrate was sintered for 2 h at 350 °C and 2 h at 550 °C and the gas sensing films with different components were parallel synthesized (see Figure 1b). Figure 1c–f show the scanning electron microscopy (SEM; Nova NanoSEM 450, FEI Company, Eindhoven, Netherlands) images with different magnifications of one modified SnO_2_ gas sensing film. There were no macroscopic and microscopic cracks. The microscopic morphology of the tin dioxide was preserved. The performance indicators of the platform were as follows. Six kinds of jettable ink could be parallel mixed into 400 premixed solutions with different components. It took two minutes to prepare a single premixed solution and three minutes to transfer print a gas sensing film.

### 2.2. High-Throughput Screening of Sensor Library

**Figure 4:**
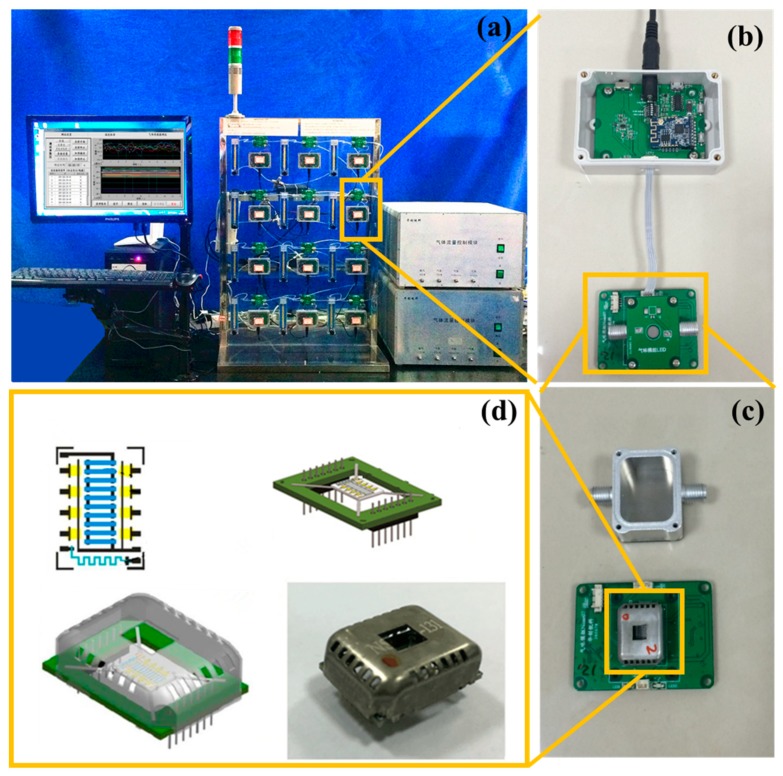
The structure of high-throughput gas sensing unmanned testing platform. (**a**) Testing platform; (**b**) Sensor module; (**c**) Testing chamber and sensor device; (**d**) The structure of the sensor device.

The next step for sensor selection and array optimization with the combinatorial and high-throughput technique is high throughput screening. A high throughput gas sensing unmanned testing platform (see Figure 4a) was designed.

The testing network consisted of sensor modules (see Figure 4b) and an automatic gas mixing device. The sensor module was in charge of gas sensing, temperature modulation, temperature and light modulation and signal acquisition. Each module can be connected with a PC through a Wi-Fi communication module respectively or as a network. For resistance testing, the schematic diagram of the sensor module resistance test was as described in our previous work [23]. After depositing the gas sensing films and sintering, the 8-matrices material substrate was packaged into a low-power gas sensor device (see Figure 4d). For resistance measuring, the sensor device with a sensor module was connected (see Figure 4c). The testing environment in the chamber of every sensor module could be regulated by the automatic gas mixing device, which could mix the target gas with the carrier gas to a specific concentration through four parallel pressure sensors, mass flow controllers and flux valves. The mixed testing gas was equally distributed into 12 pathways through rotameters. A computer cooperating with software was used to operate the sensor modules and automatic gas mixing device. The automatic signal control, acquisition and preservation of each module and gas mixing device enabled unattended testing.

The performance indicators of the platform were as follows. The measurement range of resistance was 100 Ω–1 GΩ with an error less than 5% [24,25,26,27]. The resistance acquisition rate of each channel was more than 2 Hz. According to the local area TP/IP network, the number of the sensor modules could be expanded to 254. That is to say, 2032 (8 × 254) gas sensing films could be parallel characterized. The temperature control range of the sensor module was between room temperature and 550 °C with an error less than 0.5 °C.

**Figure 5:**
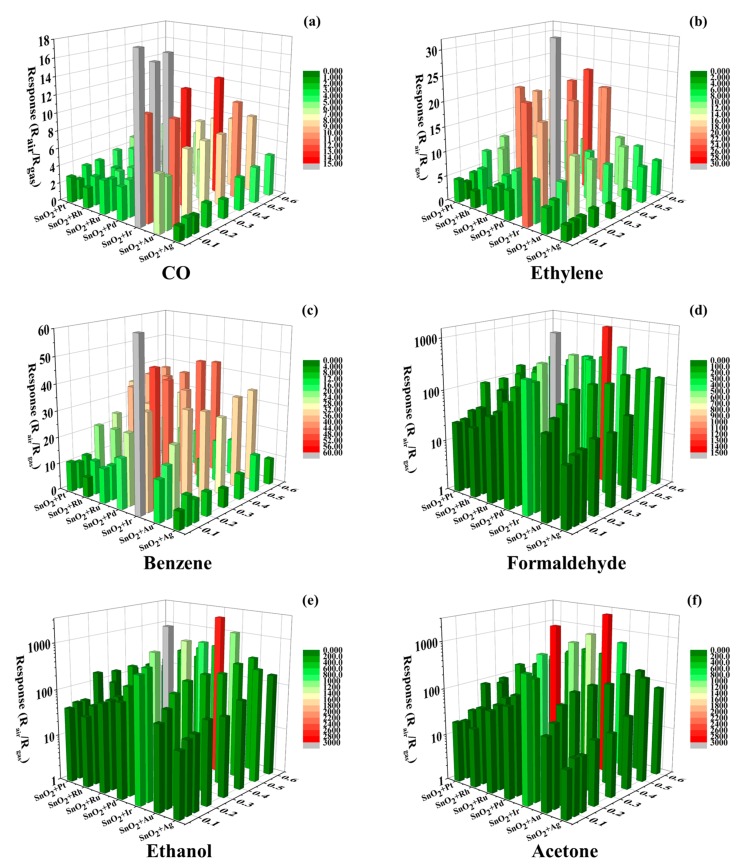
The high throughput resistance measurement (HT-RM) results of a sensor library based on nano-SnO_2_ to 100 ppm of the six tested gases at 350 °C. (**a**) CO; (**b**) Ethylene; (**c**) Benzene; (**d**) Formaldehyde; (**e**) Ethanol; (**f**) Acetone.

A high-throughput screening example of a sensor library using the platform is shown in Figure 5. The basic material of the sensor library was nano-SnO_2_. The additive materials are shown in Table 1. As can be seen, seven kinds of precious metal ions were used for surface modification. The proportion of precious metal ions added is shown in Table 2. There were fifty-five sensing films with modified precious metal ions and one pure SnO_2_ sensing film. The responses of the sensor library to CO, ethylene, benzene, formaldehyde, acetone and ethanol are shown in Figure 5. The concentration of all tested gases was 100 ppm. The tests were conducted at three constant temperature, 350 °C, 250 °C and 150 °C, respectively. According to the results, 350 °C was the optimum working temperature of all sensors. The response of all sensors at 350 °C is shown in Figure 5.

The results showed that the response to the tested gases of basic materials was improved evidently by the modified precious metal ions. The responses of pure SnO_2_ to 100 ppm to the six tested gases (CO, ethylene, benzene, formaldehyde, ethanol and acetone) were 3.0, 4.6, 14.1, 29.3, 50.0 and 19.6, respectively, while the maximum responses to the six tested gases were 20.5, 21.3, 54.1, 1452.3, 2861.5 and 2973.5, obtained by SnO_2_ + 0.05 mol% Ir, SnO_2_ + 0.2 mol% Ir, SnO_2_ + 0.05 mol% Ir, SnO_2_ + 0.5 mol% Ir, SnO_2_ + 0.5 mol% Ir and SnO_2_ + 0.5 mol% Ir, respectively. It can be concluded that the precious metal ion Ir had the largest improvement in the gas sensing performance of tin oxide. Ir modified SnO_2_ had a modest selectivity when sensing the six tested gases. This is because of the broad-spectrum response of the metal oxide gas sensitive materials. However, it could not distinguish the six tested gases based on the result shown in Figure 5.

**Figure 6:**
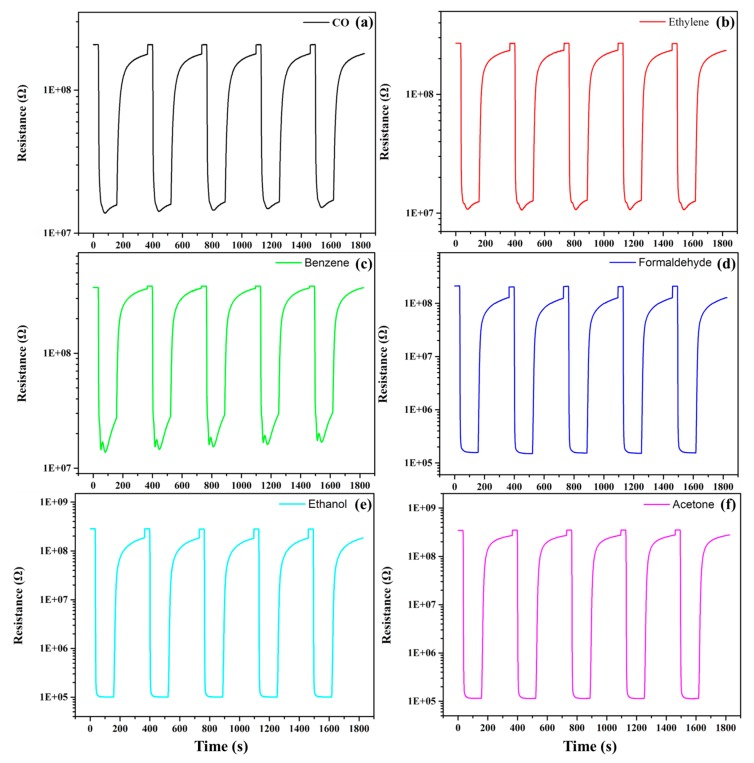
The Resistance–Time (R–T) curves of SnO_2_ + 0.5 Ir mol% to 100 ppm of the six gases at 350 °C. (**a**) CO; (**b**) Ethylene; (**c**) Benzene; (**d**) Formaldehyde; (**e**) Ethanol; (**f**) Acetone.

**Figure 7:**
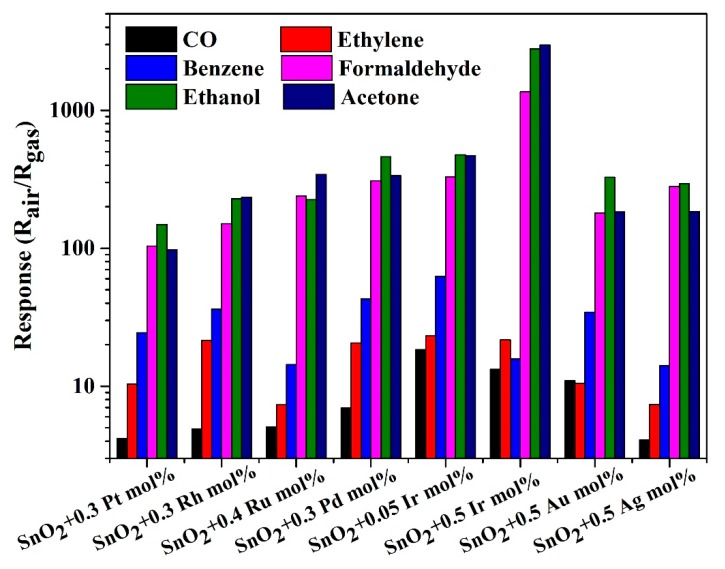
The selectivity of the eight selected materials to 100 ppm of the tested gases.

For gas identification, eight materials were selected as the optimized sensor array. The standard of material selection was as follows. First, 10 materials with a higher response of each tested gas were preliminarily selected. The preliminary selected materials were sorted according to the number of repetitions. Finally, the top eight materials were selected as the optimized materials to form array optimization. The selected eight materials were SnO_2_ + 0.3 Pt mol%, SnO_2_ + 0.3 Rh mol%, SnO_2_ + 0.4 Ru mol%, SnO_2_ + 0.3 Pd mol%, SnO_2_ + 0.05 Ir mol%, SnO_2_ + 0.5 Ir mol%, SnO_2_ + 0.5 Au mol% and SnO_2_ + 0.5 Ag mol%, respectively. To prove the accuracy of the gas sensors, the R-t (resistance-time) of one gas sensor (SnO_2_ + 0.5 Ir mol%) to the six tested gases is shown in Figure 6. The selectivity of the eight selected materials to 30 ppm tested gases is shown in Figure 7.

## 3. Details of Handheld Wireless E-Nose System

### 3.1. Handheld Wireless E-Nose System with Selected Materials 

**Figure 8:**
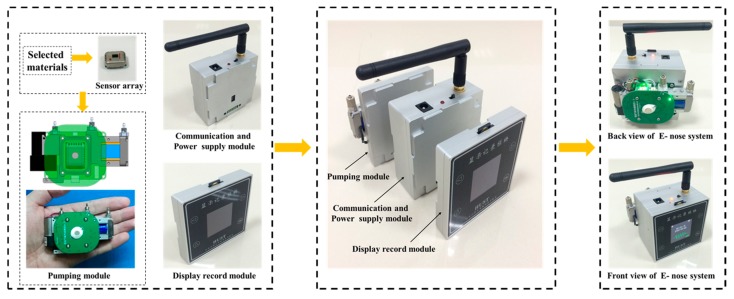
The structure of the handheld wireless E-nose system.

A handheld wireless E-nose system was designed to interface with the selected sensor array. The E-nose system mainly consisted of three parts: a pumping module, communication and power supply module and display record module, as shown in Figure 8. As can be seen, the selected materials were packed directly into the sensor device, followed by the combining sensor device and pumping module. The pumping module is in charge of the gas sensing, temperature modulation, temperature and light modulation and the environment in the testing chamber. The resistance measuring range was 100 Ω to 1 GΩ with an error less than 5%. The communication and power supply module was the power source and responsible for data acquisition and transmission and could be connected with a PC through the Wi-Fi communication module. For the display record and module, it interacted with a human through keys and the Liquid Crystal Display (LCD). The interface module also had a wireless interface with a PC for database update.

### 3.2. Application in Discrimination of Gases

The optimized sensor array (contained the selected eight materials) and handheld wireless E-nose system were combined for gas identification. Six gases with three concentrations of each gas were tested at 350 °C (optimum working temperature) by the handheld wireless E-nose system. Each sample was tested ten times. In total, 6 × 3 × 10 = 180 samples were tested. All samples were tested in a random sequence and the experiment was conducted in one week. Then, the response of each sample was extracted as the original features. The resistance response of a certain sensor to the tested gas at optimum working temperature represented the information of the reactions between them. To eliminate the testing error, two tests with the maximum and minimum fluctuations of the feature values in each sample test were excluded. A total of 28 × 8 dimensions original feature space were extracted for each sample. Then, the dimensionality of the original was reduced by principal component analysis (PCA) and the tested gases were discriminated by Fisher discriminant analysis (FDA).

**Figure 9:**
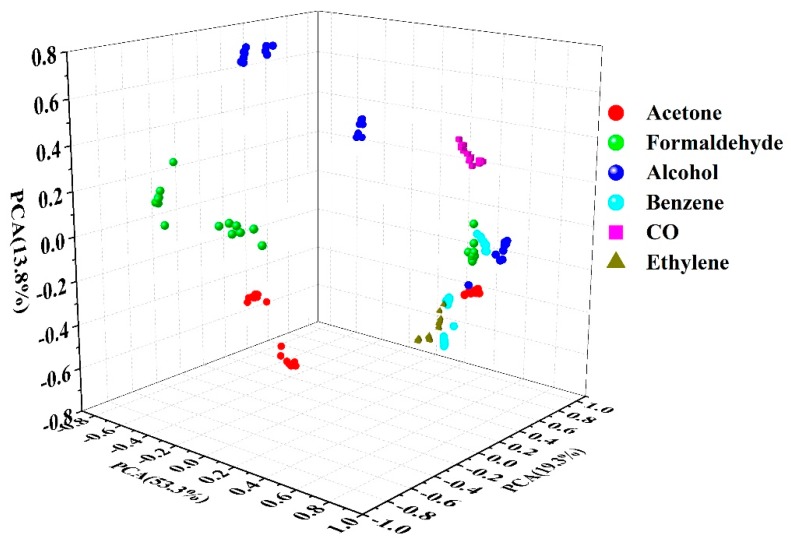
The principal component analysis (PCA) score plots of the sensing films.

**Figure 10:**
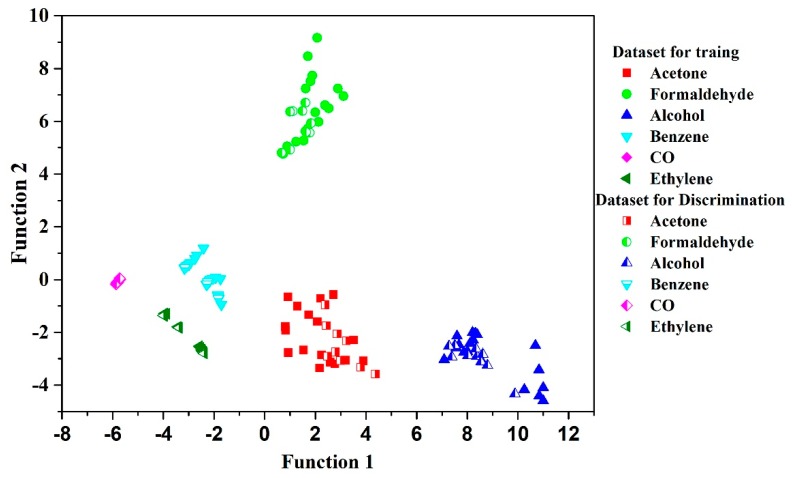
The Fisher discriminant analysis (FDA) results of all samples.

As can be seen from Figure 9, the samples of every tested gas were distributed in relatively different areas. Due to the concentration interference, part of the areas of acetone, formaldehyde, alcohol, benzene and ethylene overlapped and the distribution of acetone, formaldehyde and alcohol were not concentrated, so it could be not discriminated correctly. Furthermore, FDA was used as pattern recognition method. FDA has been widely used as a method of pattern recognition [24,25,28] and mainly constructs high-dimensional historical data into a low-dimensional principal component space. When discriminating, the data acquired in real time are projected onto the principal component space and transformed into new data for pattern recognition. The FDA can be divided into three steps. First, a category pattern vector that can express the data category is obtained. The feature vector that is the most important and sensitive feature parameter the data category is then extracted. Finally, the discriminant function composed of the feature vector is used for pattern recognition. Therefore, functions 1 and 2 are the discriminant function. In order to avoid over-fitting, K-fold cross-validation [28,29,30] was used to split the pretreated data into training and validation sets recurrently. Due to the large dataset, three-fold cross-validation was used. That is to say, the tested samples of each gas were divided into three subsamples. One subsample was used as the validation data for discrimination and the remaining two were used as training data. The FDA was repeated three times (the folds) to ensure that each of the three subsamples were used exactly once as the validation data. The two-dimensional plots of FDA are shown in Figure 10. As can be seen from Figure 10, the gas species was distributed independently. The six tested gases could be discriminated 100% correctly. The discrimination rates are shown in Table 3. The results showed that the handheld wireless E-nose system was reliable and repeatable.

## 4. Conclusions

In this study, a set of hardware platforms to improve the efficiency of new E-nose development was presented including a gas sensing film parallel synthesis platform, high-throughput gas sensing unmanned testing platform and a handheld E-nose system. Inkjet printing was used as a parallel synthesis method to parallel synthesize the sensor libraries for gas sensor selection and array optimization. A high-throughput gas sensing unmanned testing platform was designed to measure the sensor library resistances within the range 100 Ω to 1 GΩ. After array optimization among the sensors in sensor libraries, the selected sensors could be easily packaged into an eight-sensor array sensor device with low power under the working temperature from room temperature to 550 °C with an error less than 0.5 °C. A handheld wireless E-nose system was designed to interface with one sensor device. 

Fifty-five SnO_2_ sensing films with surface modification and an intrinsic SnO_2_ film were parallel deposited by the gas sensing film parallel synthesis platform. The gas sensing performance of the sensor library was obtained by the gas sensing unmanned testing platform. With the above two platforms, the screening efficiency of a suitable sensor selected from the sensor libraries was greatly improved. A portable E-nose system assembled with a suitable sensor was used for gas identification. With this set of hardware platforms, the future directions of this work include the screening efficiency of high-performance gas sensitive materials. Sensor arrays of various sensing performances will be available and can be used in many fields (food industry, environment, public safety). 

## Figures and Tables

**Table 1:** The source of additives applied in the surface modification.

Number	Element	Source	Number	Element	Source
1	Null	Null	5	Pd	PdCl_2_
2	Pt	H_2_PtCl·6H_2_O	6	Ir	IrCl_4_
3	Rh	RhCl_3_	7	Au	AuCl_3_·HCl
4	Ru	RuCl_3_·xH_2_O	8	Ag	AgNO_3_

**Table 2:** The proportion of added elements.

Number	1	2	3	4	5	6	7	8
Pt (mol%)	0	0.05	0.09	0.13	0.2	0.3	0.4	0.5
Rh, Ru, Pd, Ir, Au, Ag (mol%)	0.05	0.09	0.13	0.2	0.3	0.4	0.5	0.6

**Table 3:** The classification rates of FDA (Groups 1–6 represent CO, ethylene, benzene, ethanol, acetone and formaldehyde, respectively).

Data of Each Gas for Training	Data of Each Gas for Discrimination	Classification Rates (%)						
		Group 1	Group 2	Group 3	Group 4	Group 5	Group 6	All Samples
9–16, 17–24	1–8	100	100	100	100	100	100	100
1–8, 17–24	9–16	100	100	100	100	100	100	100
1–8, 9–16	17–24	100	100	100	100	100	100	100
The Average Classification Rates		100	100	100	100	100	100	100
